# Supplementary material for: Interictal MEG abnormalities to guide intracranial electrode implantation and predict surgical outcome
Source: ArXiv. 2023 Apr 11:arXiv:2304.05199v1. Preprint. [Version 1] (PMC10120748)
Supplement: Supplement 1 [file NIHPP2304.05199v1-supplement-1.pdf]

Supplementary

Surgical outcome separability of the D_{RS}

The D_{RS} is a measure that was recently shown to relate to post-operative seizure freedom in cohorts of individuals with refractory epilepsy[20,32]. We compute the MEG and iEEG D_{RS} values for the cohort of 32 individuals with refractory neocortical epilepsy using only tissue that has coverage in both modalities. The results across the cohort, Figure S1 demonstrate the separability of surgical outcome groups based on the derived D_{RS} scores. The D_{RS} for both modalities performs well and in the hypothesised direction, separating surgical outcome groups with an AUC> 0.7.

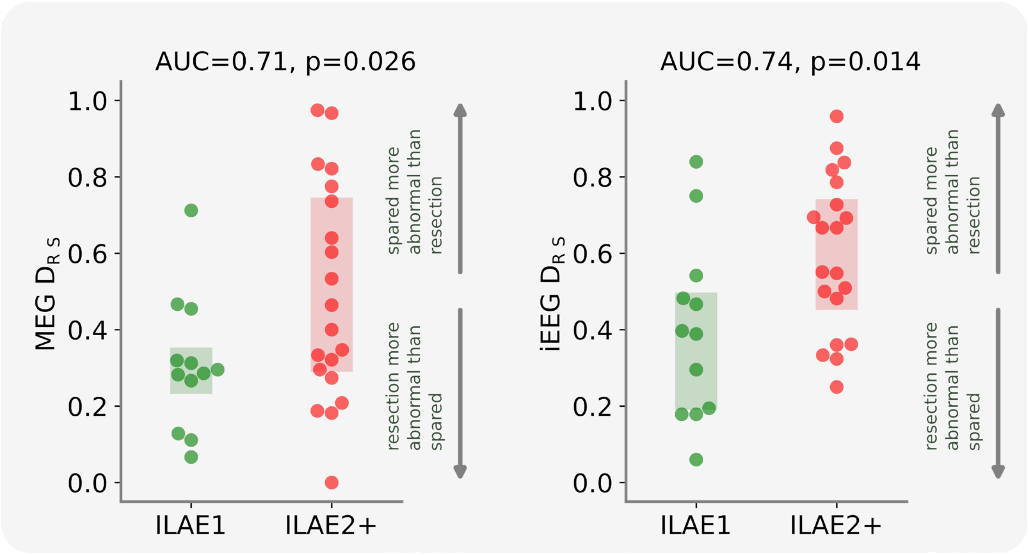

Figure S1: Surgical outcome separability of the MEG and iEEG D_{RS} measures*. Boxplots illustrated the differences between surgical outcome groups based on the corresponding D_{RS} scores. Each point corresponds to an individual, green points correspond to seizure-free individuals, and red points to non-seizure-free individuals. Both measures of D_{RS} separate surgical outcome groups well and in the hypothesised direction. That is, with smaller D_{RS} values attributed to seizure-free patients as the most abnormal tissue has been resected. Statistical significance of the AUC was calculated using a one-tailed Mann-Whitney U test.*

Table of patient data

Summary of patient metadata and measures. Commonly acquired patient metadata are reported including the side of surgical resection, localisation of the resection (F: Frontal, T: Temporal, P: Parietal, O: Occipital), one year post-operative outcome. Additionally, the abnormality coverage, and both D_{RS} derived using MEG and iEEG data are reported.

Patient ID	Side	Resection Site	Surgical Outcome (1 year)	Abnormality Coverage	iEEG D_{RS}	MEG D_{RS}
1	L	F	ILAE 2+	0.299	0.551	0.000
2	L	F	ILAE 2+	0.405	0.667	0.464
3	R	F	ILAE 2+	0.442	0.548	0.775
4	L	F	ILAE 1	0.67	0.482	0.128
5	L	F	ILAE 1	0.714	0.840	0.313
6	R	F	ILAE 2+	0.471	0.250	0.603
7	R	F	ILAE 2+	0.716	0.481	0.188
8	L	F	ILAE 2+	0.681	0.362	0.533
9	L	F	ILAE 1	0.56	0.542	0.282
10	L	F	ILAE 2+	0.532	0.694	0.833
11	L	F	ILAE 2+	0.398	0.333	0.274
12	R	P	ILAE 2+	0.608	0.838	0.347
13	L	O	ILAE 1	0.476	0.179	0.455
14	R	F	ILAE 2+	0.252	0.818	0.640
15	R	F	ILAE 1	0.586	0.397	0.467
16	R	P	ILAE 2+	0.687	0.324	0.208
17	L	T	ILAE 1	0.562	0.389	0.267
18	L	T	ILAE 2+	0.716	0.727	0.182
19	L	T	ILAE 2+	0.537	0.875	0.400
20	L	F	ILAE 1	0.63	0.060	0.295
21	L	FP	ILAE 2+	0.649	0.500	0.333
22	R	F	ILAE 2+	0.546	0.360	0.295
23	L	F	ILAE 1	0.624	0.467	0.067
24	L	F	ILAE 2+	0.508	0.786	0.821
25	L	F	ILAE 2+	0.518	0.958	0.967
26	R	OP	ILAE 1	0.826	0.194	0.319
27	R	F	ILAE 1	0.631	0.295	0.286
28	R	O	ILAE 1	0.821	0.179	0.111
29	L	OP	ILAE 2+	0.649	0.667	0.321
30	R	F	ILAE 2+	0.597	0.509	0.736
31	R	P	ILAE 2+	0.469	0.692	0.974
32	R	FP	ILAE 1	0.456	0.750	0.712
